# Effect of levosimendan infusion prior to left ventricular assist device implantation on right ventricular failure

**DOI:** 10.1186/s13019-022-01915-6

**Published:** 2022-06-16

**Authors:** Amitai Segev, Jacob Lavee, Yigal Kassif, Yedida Shemesh, Alexander Kogan, Dov Freimark, Avi Morgan, Dor Lotan, Edward Itelman, Avishay Grupper

**Affiliations:** 1 Cardiovascular Division, Sheba Medical Center, Tel Hashomer, Sheba Rd. 2, Ramat-Gan, Israel; 2 Sackler Faculty of Medicine, Tel Aviv University, Tel Aviv, Israel

**Keywords:** LVAD, Levosimendan, Left ventricular assist device, Right ventricular failure

## Abstract

**Objective:**

Investigate the safety and efficacy of preoperative levosimendan in patients undergoing left ventricular assist device (LVAD) implantation.

**Methods:**

Consecutive patients who received LVADs (HeartMate-2, 3, HVAD) in a single tertiary medical center (2012–2018). INTERMACS profile 1 patients were excluded. The primary outcome was post-LVAD right ventricular failure (RVF) and inhospital mortality rates. The secondary outcomes included other clinical, echocardiographic and hemodynamic parameters at follow-up.

**Results:**

Final cohort consisted of 62 patients (40[65%] in the levosimendan group and 22[35%] in the no-levosimendan group). Post-operative RVF rate and inotrope or ventilation support time were similar in the levosimendan and no-levosimendan groups (7.5% vs. 13.6%; *P* = 0.43, median of 51 vs. 72 h; *P* = 0.41 and 24 vs. 27 h; *P* = 0.19, respectively). Length of hospitalization, both total and in the intensive care unit, was not statistically significant (median days of 13 vs. 16; *P* = 0.34, and 3 vs. 4; *P* = 0.44, respectively). Post-operative laboratory and echocardiographic parameters and in-hospital complication rate did not differ between the groups, despite worse baseline clinical parameters in the Levosimendan group. There was no significant difference in the in-hospital and long term mortality rate (2.5% vs. 4.5%; *P* > 0.999 and 10% vs. 27.3% respectively; *P* = 0.64).

**Conclusions:**

Levosimendan infusion prior to LVAD implantation was safe and associated with comparable results without significant improved post-operative outcomes, including RVF.

**Supplementary Information:**

The online version contains supplementary material available at 10.1186/s13019-022-01915-6.

## Introduction

Left ventricular (LV) assist devices (LVADs) are an increasingly common therapy for advanced heart failure (HF). At present, LVADs represent the only available and effective alternative to heart transplantation, and provide a broad spectrum of strategies, including bridge to heart transplantation (BTT), bridge to recovery and destination therapy (DT).

Given that many patients with advanced LV dysfunction assessed for an LVAD also have some degree of right ventricular (RV) dysfunction, early RV failure (RVF) immediately after device implantation is a common complication affecting 10–40% of the patients [1].

Post LVAD implantation RVF (post-LVAD RVF) develops through multiple mechanisms, including increased venous return to the RV, decreased septal contribution to RV contraction, exacerbation of tricuspid regurgitation (TR) and tachyarrhythmias [2–5]. Clinical manifestations of post-LVAD RVF include systemic congestion, end-organ dysfunction, and/or circulatory failure [6]. Specific diagnostic criteria for RVF after CF-LVAD implantation have not been universally defined [7–9].

With accordance to previous studies, including the pivotal MOMENTUM-3 trial [10],post-LVAD RVF was considered as a need for right ventricular assist device (RVAD) support or inotrope support for longer than 7 days after the surgery [7, 11].

Post-LVAD RVF confers worse prognosis and constitutes a major cause of morbidity and mortality in the early post-operative period [12, 13].

Levosimendan is an inodilator that increases cardiac contractility via calcium sensitization [14]. Several studies investigating the effect of perioperative levosimendan in patients undergoing cardiac surgery did not show a clear clinical benefit, and the effect of levosimendan in the context of RVF is still unclear [15–17].

There is very little data on the safety and efficacy of preoperative levosimendan infusion in patients undergoing LVAD implantation. The objective of this study was to investigate the effect of preoperative levosimendan administration on post-operative RVF and other clinical, laboratory, echocardiographic and hemodynamic outcomes.

## Patients and methods

A retrospective analysis of all consecutive patients who received continuous flow LVADs (Heartmate 2, 3 or HVAD) between August 2012 and May 2018 at a single tertiary medical center. All patients underwent a comprehensive evaluation before implantation according to local protocols, perioperative hospitalization in the cardiac surgery department and routine follow-up post-implantation at the LVAD clinic in our heart failure institute. Patients classified as INTERMACS 1 (cardiogenic shock) [18] prior to LVAD implantation and patients who were implanted a biventricular assist (BIVAD) device were excluded from the study.

The study was approved by the Institutional Ethics Committee. Consent of patients has been waived. The data underlying this article will be shared on reasonable request to the corresponding author.

Pre and post-operative data (demographic, clinical, echocardiographic, hemodynamic and laboratory) for each patient were abstracted from the medical records. Of note, most pre-operative right heart studies were performed after medical optimization, including levosimendan administration. CKD was defined as estimated creatinine clearance of less than 60 mL/min/1.73 m^2^ calculated by the MDRD formula. Pre-operative echocardiographic examinations were performed at 5 (range 1–149) days and hemodynamic catheterizations were performed at 41 (range 1–220) days before surgery. Post-operative echocardiographic examinations were performed during the index hospitalization and at 14 (range 0–117) days and hemodynamic catheterizations were performed at 6 (range 1.3–13.1) months after surgery. Post-operative complications were defined according to the society of thoracic surgeons definitions for post coronary artery bypass graft surgeries including deep sternal wound infection, bleeding, renal failure, surgical re-exploration, prolonged ventilation and stroke [19]. We also included other relevant complications including ventricular or atrial arrhythmia and postpericardiotomy syndrome (PPS). As previously mentioned, RVF was defined as inotropic support for over 7 days.

Patients were stratified into two groups based on preoperative levosimendan infusion (levosimendan group vs. no-levosimendan group). Up to November 2016 the decision whether to administer preoperative levosimendan was left to the physician discretion. After that point in time, all patients received preoperative levosimendan as part of an institutional protocol.

Preoperative, intraoperative and post-operative clinical, laboratory, echocardiographic and hemodynamic parameters were compared between the groups.

### Statistical analysis

Categorical variables were described as frequency and percentage and continuous variables as median and interquartile range (IQR). Continuous variables were compared between the two groups using Mann–Whitney Test and categorical variables were compared using Chi-Square Test of Fischer’s Exact Test.

Length of follow up was observed using reversed censoring method. Kaplan–Meier curve was used to describe events (mortality, transplant, complications etc.) during the follow up period, and Log-Rank test was used to compare between the groups. Multivariable analysis was performed using Cox Regression Model for events with a known duration to the event and a General Binomial Logistic Regression for events without an unknown time to event.

All statistical tests were two sided and *P* < 0.05 was considered as statistically significant.

SPSS was used for all statistical analysis (IBM SPSS Statistics, version 25, IBM corp, Armonk, NY, USA, 2017).

## Results

### Patient characteristics

The study cohort consisted of 62 patients (88.7% males) who underwent LVAD implantation, after exclusion of patients with an INTERMACS 1 score prior to implantation surgery (n = 2), patients who underwent a BIVAD implantation (n = 4), patients with both (n = 2) and patients with missing data regarding preoperative levosimendan treatment (n = 1). No race-based differences were present.

The median follow-up period was 21 months (IQR 14–31).

Patients were implanted with a HeartMate 2 device (implantations up to January 1st 2016, n = 20), HeartMate 3 device (implantations after January 1st 2016, n = 39), or a HeartWare device (n = 3).

The main HF etiology in both groups was ischemic cardiomyopathy followed by idiopathic dilated cardiomyopathy. The majority of patients in both groups were implanted as part of a BTT (total of 90.3%). All patients were severely symptomatic (Table 1).

**Table 1:** Characteristics of the patients at baseline

Characteristic	Pre-op levosimendan (n = 40)	No-levosimendan (n = 22)	Total (n = 62)	*P* value
Age—yr	57.3 (51.3–62.9)	57.2 (52.6–65.1)	57.2 (51.7–63.1)	0.79
Male sex	33 (82.5)	22 (100)	55 (88.7)	0.04
HF etiology				0.16
ICMP	18 (45)	14 (63.6)	32 (51.6)	
DCM	21 (52.5)	7 (31.8)	28 (45.1)	
NYHA class				0.003
3B	2 (5)	8 (36.4)	10 (16.1)	
4	38 (95)	14 (63.6)	52 (83.9)	
INTERMACS score				0.08
2	17 (42.5)	6 (27.3)	23 (37.1)	
3	9 (22.5)	2 (9.1)	11 (17.7)	
4	14 (35)	14 (63.6)	28 (45.2)	
Diabetes mellitus	17 (42.5)	9 (40.9)	26 (41.9)	0.9
Chronic kidney disease	13 (32.5)	11 (50)	24 (38.7)	0.17
Creatinine—mg/dL	0.98 (0.73–1.39)	1.3 (1.09–1.6)	1.15 (0.83–1.44)	0.01
MDRD—mL/min/1.73 m^2^	78.4 (55.9–105.9)	61.5 (47.5–76.0)	68.2 (52.4–97.9)	0.029
Bilirubin—mg/dL	0.8 (0.56–1.17)	0.86 (0.68–1.7)	0.8 (0.62–1.24)	0.27
Albumin—g/dL	3.7 (3.4–4.0)	4 (3.6–4.4)	3.8 (3.4–4.1)	0.024
Echocardiography				
EF—%	15 (10–20)	17.5 (12.2–25.0)	15 (10–20.5)	0.27
MR—above moderate	18 (45)	7 (31.8)	25 (40.3)	0.31
TR—above moderate	4 (10)	3 (13.6)	7 (11.3)	0.69
Enlarged RV	18 (40)	7 (31.8)	25 (40.3)	0.31
RV function				0.15
Normal	6 (15)	8 (36.4)	14 (22.6)	
Mild reduction	13 (32.5)	5 (22.7)	18 (29)	
Moderate–severe reduction	21 (52.5)	9 (40.9)	30 (48.4)	
SPAP—mmHg	55 (50.25–60)	53 (41–60)	54.5 (48.75–60)	0.55
Right heart catheterization				
CO—l/min	2.93 (2.48–3.67)	3.19 (2.53–3.69)	2.98 (2.5–3.68)	0.48
PCWP—mmHg	28 (23.75–31.5)	23.5 (18.75–30.25)	27 (21.25–31)	0.047
mPA—mmHg	41.5 (34.5–46)	33 (30.5–46.5)	40 (33–46)	0.32
mRA—mmHg	10 (7–13)	8 (4–16.5)	9.5 (6–13)	0.56
RA/PCWP	0.37 (0.27–0.47)	0.37 (0.25–0.46)	0.37 (0.25–0.46)	0.98
PAPi	3.75 (2.79–5.21)	4.75 (3.15–12)	4.5 (2.8–10.7)	0.21
PVR—wood units	3.17 (2.42–4.87)	4.02 (2.19–6.13)	3.41 (2.4–5.16)	0.43

Data are presented as median (Interquartile range) or number (%)

ICMP, ischemic cardiomyopathy; IDC, idiopathic dilated cardiomyopathy; SPAP, systolic pulmonary artery pressure; CO, cardiac output; PCWP, pulmonary capillary wedge pressure; mPA/RA, mean pulmonary artery/right atrium pressure; PAPi, pulmonary artery pulsatility index; PVR, pulmonary vascular resistance

The levosimendan and no-levosimendan groups did not differ in the baseline prevalence of ventricular or atrial tachyarrhythmias (62.5% vs. 41%; *P* = 0.1 and 47.5% vs. 60%; *P* = 0.38, respectively), the presence of an ICD or CRT apparatus (75% vs. 91%; *P* = 0.18 and 42.5% vs. 54.5%; *P* = 0.36, respectively) and the concurrent antiarrhythmic or digitalis therapy (60% vs. 54.5%; *P* = 0.67 and 37.5% vs. 50%; *P* = 0.34, respectively).

Baseline echocardiographic parameters were similar between the groups. The mean left ventricular ejection fraction (EF) was 15% (IQR 10–20.5). Almost half of the patients were considered to have at least moderate RV dysfunction (48.4%, n = 30) and 11.3% (n = 7) above moderate TR.

Hemodynamic data at baseline showed increased mean right atrial (mRA) and pulmonary (mPA) pressure in both groups. Despite elevated pulmonary capillary wedge pressure (PCWP) in the levosimendan group, both the ratio between RA to PWCP (RA/PCWP) and the pulmonary artery pulsatility index (PAPi) were similar between the groups. The levosimendan group had a significantly increased proportion of NYHA 4 patients and lower estimated creatinine clearance levels, compared to the no-levosimendan group.

### Short-term post-operative outcomes

Thirteen patients (21%) underwent a total of 17 additional surgical procedures during the LVAD surgery, including aortic valve replacement (n = 6), tricuspid valve replacement (n = 7), patent foramen ovale closure (n = 3) and mitral clip removal (n = 1), with similar rates between the groups (Table 2). LVAD speed, measured at day one after surgery and adjusted as standard deviation from average speed for each device, was similar in both groups (− 0.15, IQR [− 0.1]–[− 0.66] in the levosimendan group vs. − 0.1, IQR [− 0.12]–[− 0.54] in the no-levosimendan group; *P* = 0.36). There was no difference in the post-operative inotrope and ventilation support time (median of 51 h vs. 72 h; *P* = 0.41 and 24 h vs. 27 h; *P* = 0.19, respectively), as well as the maximal central venous pressure (CVP) post-surgery (22 mmHg, IQR 17.75–26.25 vs. 23 mmHg, IQR 19–26; *P* = 0.58). There was also no difference in the number of inotropic drugs used, as three or more drugs were used at a similar rate in both groups (26% in the levosimendan group vs. 12% in the no-levosimendan group; *P* = 0.41).

**Table 2:** Short-term post-operative outcomes

Outcome	Pre-op levosimendan (n = 40)	No-levosimendan (n = 22)	Total (n = 62)	*P* value
Additional surgical procedures	10 (25)	3 (13.6)	13 (21)	0.34
Cardiopulmonary bypass time—minutes	93 (83.75–115.25)	105 (87.5–127.5)	97.5 (84.25–116.75)	0.39
Post-op ventilation support time—hours	24 (22–29.75)	27 (22–56.75)	25 (22–48)	0.19
Maximal CVP—mmHg	22 (18–26)	23 (19–26)	22 (19–26)	0.58
Post-op inotrope support time—hours	51 (19–88)	72 (18–125)	60 (19–112)	0.41
Inotropic support > 14 days	1 (2.5)	1 (4.5)	2 (3.2)	0.66
RVF	3 (7.5)	3 (13.6)	6 (9.6)	0.43
Echocardiography at discharge				
Enlarged RV	14 (35)	9 (40.9)	23 (37.1)	> 0.999
RV function				0.59
Normal	7 (24.1)	5 (29.4)	12 (19.4)	
Mild reduction	4 (13.8)	4 (23.5)	8 (12.9)	
Moderate—severe reduction	18 (62.1)	8 (47.1)	26 (41.9)	
Laboratory at discharge				
Creatinine—mg/dL	0.77 (0.63–1.02)	0.99 (0.76–1.09)	0.87 (0.69–1.09)	0.073
MDRD—mL/min/1.73 m^2^	96.3 (78.4–143.3)	83.8 (74.7–111.2)	95.7 (78.1–132.1)	0.19
Bilirubin—mg/dL	0.61 (0.44–0.9)	0.68 (0.5–0.86)	0.87 (0.69–1.09)	0.39
Albumin—g/dL	3 (2.8–3.2)	2.9 (2.6–3.1)	2.9 (2.7–3.2)	0.31
Post-op Complications—Any	15 (68.1)	21 (52.5)	36 (58)	0.23
Total hospitalization length –days	13 (9.25–23.5)	16 (10.75–25.75)	14 (10–24.25)	0.53
ICU hospitalization length—days	3 (2–5)	4 (2–6)	3.5 (2–6)	0.44
Short term mortality				
During hospitalization	1 (2.5)	1 (4.5)	2 (3.2)	> 0.999
30 days	0 (0%)	1 (4.5%)	1 (1.6)	0.76

Data are presented as median (Interquartile range) or number (%)

ICU = intensive Care Unit; CVP = central venous pressure; RVF = right ventricular failure

No patient in either group required RVAD support. The rate of post-LVAD RVF, defined as inotrope support for longer than 7 days, was similar between the levosimendan and no-levosimendan groups (7.5% vs. 13.6% respectively; *P* = 0.43). Rate of inotropic support for more than 14 days was also similar between the groups (2.5% vs. 4.5% respectively; *P* = 0.66).

Echocardiographic parameters before discharge were similar between the groups. The incidence of valvular abnormalities including the presence of above moderate aortic, mitral or tricuspid regurgitation did not differ between the groups (2.8% vs. 4.5%, 7.1% vs. 9.1%, 3.1% vs. 12.5% respectively; *P* > 0.1 for all). There was however a higher systolic pulmonary artery pressure (SPAP) in the levosimendan treated group (55 mmHg, IQR 50.2–60) compared to the control group (39 mmHg, IQR 37–49; *P* = 0.004).

The median hospitalization time, both total and in the ICU, was similar between the groups. There was no statistically significant difference between the two groups in the rates of total or individual post-operative complications including deep sternal wound infection (no cases in both groups), bleeding (0% vs. 4.5%; *P* = 0.35), renal failure (2.5% vs. 4.5%; *P* > 0.99), surgical re-exploration (10% vs. 22.7%; *P* = 0.25), prolonged ventilation (2.5% vs. 0%; *P* > 0.99), stroke (no cases in both groups), pneumonia (7.5% vs. 13.6%; *P* = 0.65), ventricular or atrial arrhythmia (10% vs. 4.5%; *P* = 0.64 and 12.5% vs. 13.6%; *P* > 0.99) and PPS (7.5%vs. 4.5%; *P* > 0.99, respectively).

The mortality rate during the hospitalization and at 30 days after the surgery were similar in the levosimendan and no-levosimendan groups (2.5% vs. 4.5%; *P* > 0.99 and 0% vs. 4.5%; *P* = 0.76, respectively).

### Long-term post-operative outcomes

Right heart studies performed at an average of 6 months after surgery (range 1.3–13.1) showed a lower CO and higher mRA pressure in the levosimendan group (Table 3).

**Table 3:** Long-term post-operative outcomes

Outcome	Pre-op levosimendan (n = 40)	No-levosimendan (n = 22)	Total (n = 62)	P-value
NYHA class at 3 months				0.73
1–2	32 (82.1)	16 (76.2)	48 (77.4)	
3–4	7 (17.9)	5 (23.8)	12 (19.4)	
Laboratory at 3 months				
Creatinine—mg/dL	0.9 (0.75–1.17)	0.94 (0.83–1.1)	0.93 (0.8–1.13)	0.54
Bilirubin—mg/dL	0.61 (0.44–0.9)	0.61 (0.5–0.74)	0.59 (0.49–0.72)	0.47
Albumin—g/dL	3 (2.8–3.2)	3.4 (3.3–3.7)	3.9 (3.4–4.1)	0.02
Right heart catheterization				
CO—l/min	4 (3.5–4.4)	4.4 (4.2–5.3)	4 (3.6–4.5)	0.02
PCWP—mmHg	11 (8–17)	9 (5–14)	11 (8–15)	0.15
mPA—mmHg	23 (18–27)	19 (14–24)	21 (18–27)	0.21
mRA—mmHg	11 (8–14)	5 (3–9)	10 (6–13)	0.004
PVR—wood units	2.2 (1.7–3)	1.8 (1.7–1.9)	1.9 (1.7–2.7)	0.52
Readmissions				
14 days	6 (15.4)	3 (14.3)	9 (14.5)	> 0.99
3 months	7 (17.9)	5 (23.8)	12 (19.4)	0.73
1 year	27 (69.2)	13 (61.9)	40 (64.5)	0.56
Long-term complications—Any	20 (50)	9 (40.9)	29 (46.8)	0.46
Long term mortality—no				
60 days	1 (2.5%)	1 (4.5%)	2 (3.2)	> 0.99
90 days	1 (2.5%)	1 (4.5%)	2 (3.2)	> 0.99
Total	4 (10)	6 (27.3)	10 (16.1)	0.64

Data are presented as median (Interquartile range) or number (%)

RBC, red blood cells; CVP, central venous pressure; ICU, intensive care unit

There was a similar rate of total and individual long term complications in the levosimendan and no-levosimendan groups including infection (35% vs. 13.6%; *P* = 0.08), bleeding (15% vs. 36.4%; *P* = 0.09), thrombotic (5% vs. 9.1%; *P* = 0.67) and device technical malfunction events (0% vs. 4.5%; *P* = 0.18, respectively).

**Fig. 1:**
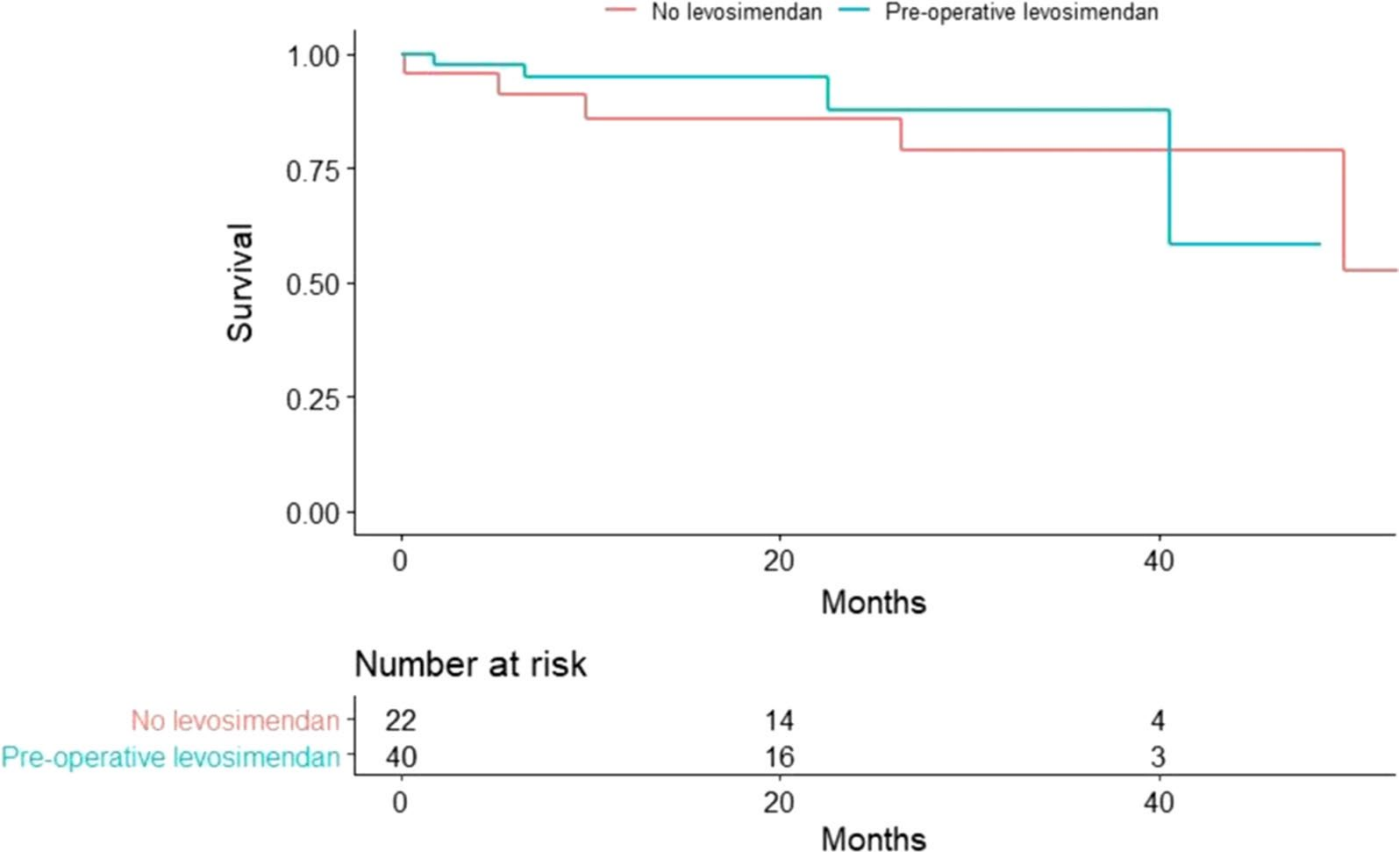
Overall survival—levosimendan group vs. no-levosimendan group

The overall mortality rate throughout the follow-up period did not differ between the groups (10% vs. 27.3%; *P* = 0.64) (Fig. 1).

### Subanalysis of patients who received preoperative levosimendan before institutional protocol

Since preoperative levosimendan became a part of our institutional protocol during the study follow up period, we performed a subgroup analysis of patients who received preoperative levosimendan before it became part of the protocol (“group A”; n = 13) and compared them to those who were not treated with the drug during the same period (“group B”; n = 22) and to those who received the drug routinely thereafter (“group C”; n = 27). Most of the clinical, laboratory and hemodynamic parameters were not statistically different between the groups, except for a higher proportion of NYHA 4 patients in group A compared to group B and more INTERMACS 2 patients in group A compared to both groups (see Additional file 1: Table S1).

### Multivariable analysis

In order to account for baseline differences between the levosimendan and no-levosimendan groups we performed a multivariable analysis that included age, NYHA and INTERMACS scores and baseline kidney functions. Even after adjustment for these variables, preoperative treatment with levosimendan was not associated with lower rates of mortality or post-LVAD RVF (see Additional file 1: Table S2 and S3, respectively). Due to low event rates, we were unable to perform a multivariable analysis on determinants associated with inotropic support for over 14 days.

## Discussion

This is the largest study thus far to our knowledge to evaluate the clinical utility of levosimendan administration in patients awaiting LVAD implantation. In our study levosimendan infusion prior to LVAD implantation was safe, well tolerated and was not associated with increased adverse events. On the other hand, it was not associated with significant improved short or long term post-operative outcomes, including post-LVAD RVF.

Post-LVAD RVF is a common complication that confers worse prognosis. RVF or multisystem organ failure was found to be the leading cause of early mortality (30-day or during the index hospitalization) among patients after LVAD implantation [12, 13].

Levosimendan is an inodilator developed in the 1990s that increases cardiac contractility via calcium sensitization, promotes vasodilatation by opening of ATP-dependent potassium channels in vascular smooth muscle cells and mediates a cardioprotective effect by opening of ATP-dependent potassium channels in cardiomyocytes’ mitochondria which in turn inhibits myocyte apoptosis [20].

Levosimendan is administered as a 24 h infusion and exhibits a rapid onset of action with maximal therapeutic effects occurring at days 1–3 post-infusion and extended cardiovascular therapeutic effect sustained for 2–3 weeks due to its long-acting active metabolites [14].

Several studies have evaluated the efficacy and safety of levosimendan in patients with HF. Despite improvement in indices of cardiac performance and HF, there is no clear evidence of short- or long-term clinical benefit [21–24]. Levosimendan is currently approved for intravenous use in some countries in Europe and South America, but remains investigational in the United States [21].

The effect of levosimendan in the context of RVF is unclear. A meta-analysis of 6 randomized controlled trials evaluating the efficacy and safety of levosimendan in patients with acute RVF with a variety of heart and lung diseases showed significant changes in echocardiographic measurements. Nonetheless, adverse events did not significantly improve following levosimendan treatment [15].

The role of perioperative levosimendan in patients undergoing cardiac surgery have been investigated in several studies with indications emerging that it can reduce the risk of low-output cardiac syndrome [25]. In a recent meta-analysis levosimendan use before cardiac surgery was associated with reduced mortality, low-output cardiac syndrome incidence and acute kidney injury events [26].

Data on the use of levosimendan as preoperative treatment before LVAD implantation surgery is scarce. In a study published in 2012 [27], Sponga et al. examined the hemodynamic and prognostic effect of levosimendan infusion in patients with borderline RV function before urgent LVAD implantation. The study included 21 patients admitted to the ICU before LVAD implantation due to severe refractory HF with evidence of impending multiorgan failure. No relevant side effects were documented, including arrhythmia, tachycardia or hypotension. Hemodynamic parameters have improved 48 h after levosimendan infusion: cardiac index increased by 21% (*P* = 0.014), PAP decreased by 12% (*P* = 0.003), PCWP and CVP both decreased by 15% (*P* = 0.028 and *P* = 0.016 respectively) and mixed venous oxygen saturation increased (*P* = 0.008). Despite the beneficial effects on RV hemodynamics prior to LVAD implantation surgery, levosimendan treatment did not prevent post-operative RVF, which occurred in 19% of the patients. The survival rate was 86% at 30 days and 57% at 1 and 2 years following LVAD support.

A later study [28] performed a post hoc analysis of 9 patients receiving levosimendan treatment pre-LVAD implantation surgery. All patients were classified as INTERMACS 2 due to deterioration of renal and hepatic function under inotropic therapy. Application of levosimendan was safe and 12-month survival rate was 89%. Two patients (22.2%) required post-operative temporary extracorporeal membrane oxygenation support due to intraoperative RVF. Data on other post-operative complications and readmission rate were not reported in this study.

Compared with the previous studies mentioned, our cohort included a larger number of patients and had a control group. The vast majority of patients in our study had RV function impairment before surgery, and almost 50% had at least moderate RVF per echo. Impaired RV function at baseline may have led to selection bias, and thus undermine any beneficial effect on RV function by levosimendan. However, we found a relatively low rate of post-LVAD RVF and mortality in our study, compared to the data cited in the literature above, rendering the last argument questionable. A possible explanation to the low event rate is the fact that most patients were hospitalized prior to LVAD implantation surgery, were medically optimized with inotrope and diuretic treatment and lacked clinical evidence of RVF at the time of surgery.

Another possible explanation to levosimendan’s lack of efficacy shown in our study is the difference in patients’ baseline characteristics between the groups, but even after adjusting for these variables, treatment with levosimendan was not associated with lower rates of mortality or post-LVAD RVF. Of note, both RA/PCWP ratio and PAPi were not significantly different between the two groups at baseline, further supporting their hemodynamic similarity. In a subgroup analysis we found that patients treated with levosimendan before it became the standard of practice at our institution were clinically more ill, as manifested by a higher proportion of NYHA 4 and INTERMACS 2 patients, and therefore were considered for an additional therapy to improve postoperative outcomes. The fact that despite worse clinical status at baseline the patients had similar outcomes might suggest a possible benefit of levosimendan therapy. Nevertheless, adjusted multivariable analysis did not support an independent association between levosimendan therapy and better clinical outcomes. Overall, despite a high proportion of critically ill patients with a substantial RV dysfunction at baseline in our study, we hereby report comparatively excellent results, including short hospitalization length and low post-LVAD RVF and mortality rate [1, 12, 27]. These observations including small sample size and low event rate of RVF and mortality in the patient cohort, may at least in part explain the lack of efficacy levosimendan exhibited in our study.

It is also important to mention the lack of adverse events including arrhythmias among the levosimendan treated patients compared with the no-levosimendan group. Due to the lack of adverse events on the one hand, in the face of a numerical improvement of outcomes in the levosimendan group and overall low event rates of mortality and post-LVAD RVF on the other, we maintained our policy of routine levosimendan administration before LVAD surgery at our institution. Our study can be thus seen as hypothesis forming and used as a foundation for further research.

We acknowledge some limitations to our study. First, this was an observational study with a retrospective analysis of collected data. Second, the study was conducted in a single tertiary medical center and there may have been patient selection bias. Third, as previously mentioned pre-operative treatment with levosimendan in our institution became the standard of practice towards the end of the study period. Of note, inotropic support for over 14 days was not included in the multivariable analysis due to the paucity of events in both groups. In addition, the decision whether to treat patients with other inotropes before the LVAD implantation was at the physician’s discretion and was not based on institution protocol. Therefore, potential confounders in that regard were not accounted for. Lastly, despite numerical differences in favor of levosimendan use, the small patient cohort and low event rate of post-LVAD RVF may lack the statistical power to identify significant differences in the outcomes measured. Nevertheless, this is the largest cohort addressing this clinical issue thus far.

## Conclusion

In conclusion, in this study we have assessed the largest cohort of advanced HF patients treated with levosimendan prior to LVAD implantation surgery. Post-LVAD outcomes were comparable between patients with or without preoperative levosimendan, despite worse clinical parameters in the levosimendan group. Levosimendan infusion, although safe, was not associated with improved post-operative clinical, laboratory, echocardiographic or hemodynamic parameters. Future larger, randomized controlled prospective studies are warranted.

## Supplementary Information

**Additional file 1.** Supplementary material.

## Data Availability

The data underlying this article will be shared on reasonable request to the corresponding author.
