# Supplementary material for: Effect of levosimendan infusion prior to left ventricular assist device implantation on right ventricular failure
Source: J Cardiothorac Surg. 2022 Jun 16;17:158. doi: 10.1186/s13019-022-01915-6 (PMC9205013; doi:10.1186/s13019-022-01915-6)
Supplement: Supplementary file 1 — Additional file 1. Supplementary material. [file 13019_2022_1915_MOESM1_ESM.docx]

| Supplementary Table 1. Subgroup analysis of patients who received preoperative levosimendan before institutional protocol | | | | | |
| --- | --- | --- | --- | --- | --- |
| Characteristic | **Group A (n = 13)** | **Group B (n = 22)** | **Group C (n = 27)** | **Total (n=62)** | **P value** |
| Age - yr | 54.6 (49.4 - 58.1) | 57.2 (53.3 -64.3) | 58.8 (52.9 - 63.2) | 57.2 (51.7 - 63.1) | 0.28 |
| Male sex | 12 (92.3) | 22 (100) | 21 (77.7) | 55 (88.7) | 0.045 |
| ICMP | 5 (38.5) | 14 (63.6) | 13 (48.1) | 32 (51.6) | 0.31 |
| Destination therapy | 1 (7.7) | 2 (9.1) | 3 (11.1) | 6 (9.7) | 0.93 |
| Class 4 NYHA | 11 (84.6) | 14 (63.6) | 27 (100) | 52 (83.9) | 0.003 |
| INTERMACS score  2  3  4 | 9 (69.2)  1 (7.7)  3 (23.1) | 6 (27.3)  2 (9.1)  14 (63.6) | 8 (29.6)  8 (29.6)  11 (40.8) | 23 (37.1)  11 (17.7)  28 (45.2) | 0.021 |
| Diabetes Mellitus | 4 (30.8) | 9 (40.9) | 13 ( 48.1) | 26 (41.9) | 0.57 |
| Chronic kidney disease | 0 (0) | 4 (18.2) | 4 ( 14.8) | 24 (38.7) | 0.27 |
| Creatinine - mg/dL | 1.3 (0.88 - 1.46) | 1.3 (1.1 - 1.59) | 0.89 (0.7 - 1.33) | 1.15 (0.83 – 1.44) | 0.013 |
| MDRD - mL/min/1.73 m² | 60.3 (54.08 - 107.1) | 61.52 (47.7 - 73.8) | 85.3 (59.9 - 102.47) | 68.2 (52.4 - 97.9) | 0.061 |
| Bilirubin - mg/dL | 0.99 (0.6 - 1.2) | 0.86 (0.7 - 1.6) | 0.79 (0.5 - 1.06) | 0.8 (0.62 - 1.24) | 0.36 |
| Albumin - g/dL | 3.4 (3.1 - 3.7) | 4.0 (3.7 - 4.3) | 3.8 (3.5 - 4) | 3.8 (3.4 - 4.1) | 0.016 |
| Echocardiography |  |  |  |  |  |
| EF – %, median (IQR) | 15 (10 – 15) | 17.5 (13.5 - 25) | 15 (15 - 20) | 15 (10 - 20.5) | 0.13 |
| AR  No – mild  Mild – severe | 11 (84.7)  2 (15.3) | 20 (90.9)  2 (9.1) | 25 (92.6)  2 (7.4) | 56 (90.3)  6 (9.7) | 0.79 |
| MR - above moderate | 3 (23.1) | 7 (31.8) | 15 (55.5) | 25 (40.3) | 0.27 |
| TR – above moderate | 1 (7.7) | 3 (13.6) | 3 (11.1) | 7 (11.3) | 0.81 |
| Enlarged RV | 5 (38.5) | 7 (31.7) | 13 (48.1) | 25 (40.3) | 0.36 |
| RV function  Normal  Mild reduction  Moderate–severe reduction | 2 (15.4)  4 (30.8)  7 (53.8) | 8 (36.4)  5 (22.7)  9 (40.9) | 4 (14.8)  9 (33.3)  14 (51.9) | 14 (22.6)  18 (29)  30 (48.4) | 0.48 |
| SPAP- mmHg, | 53 (50.5 - 55.5) | 53 (42.75 - 59.5) | 56 (52 - 61) | 54.5 (48.75 - 60) | 0.47 |
| Right Heart Catheterization | | | | | |
| CO – l/min | 2.68 (2.5 - 2.98) | 3.19 (2.57 - 3.65) | 2.99 (2.5 - 3.77) | 2.98 (2.5 - 3.68) | 0.58 |
| PCW - mmHg | 30 (27.5 - 36) | 23.5 (19 - 29.5) | 27 (23 - 31) | 27 (21.25 - 31) | 0.031 |
| mPA – mmHg | 47 (40.5 -49) | 33 (31 -46) | 40 (33 -43) | 40 (33 - 46) | 0.039 |
| mRA - mmHg | 11 (7.5 - 16.5) | 7 (2 - 11) | 7 (4.5 - 9.5) | 9.5 (6-13) | 0.675 |
| PVR - wood units | 4.4 (3.65 - 5.73) | 3.02 (2.45 - 4.11) | 2.31 (2.07 - 2.43) | 3.41 (2.4 - 5.16) | 0.042 |
| Data are presented as median (interquartile range) or number (%). Group A = patients who received preoperative levosimendan before it was part of an institutional protocol (before Nov. 2016); Group B = patients who did not receive preoperative levosimendan during the same period; Group C = patients who received preoperative levosimendan routinely thereafter (after Nov. 2016); IQR=Interquartile range; ICMP=Ischemic Cardiomyopathy; NYHA=New York Heart Association; SPAP=Systolic Pulmonary Artery Pressure; CO= Cardiac Output; PCW= Pulmonary Capillary Wedge Pressure; mPA= Mean Pulmonary Artery Pressure; mRA= Mean Right Atrium Pressure; PVR=Pulmonary Vascular Resistance | | | | | |

| Supplementary Table 2. Determinants of mortality | | | |
| --- | --- | --- | --- |
|  | **HR** | **95% CI** | **P** |
| Age | 1 | 0.93 - 1.08 | 0.972 |
| NYHA Class 4 | 0.82 | 0.09 -7.64 | 0.862 |
| INTERMACS Score 2 | 2.5 | 0.45 - 14.02 | 0.297 |
| Baseline eGFR | 0.99 | 0.97 - 1.02 | 0.478 |
| Pre-op levosimendan | 0.79 | 0.17 - 3.58 | 0.756 |
| Multivariate cox regression model on determinants associated with mortality.  CI=confidence interva; HR=hazard ratio; eGFR= estimated glomerular filtration rate, using MDRD equation | | | |

| Supplementary Table 3. Determinants of right ventricular failure | | | |
| --- | --- | --- | --- |
|  | **HR** | **95% CI** | **P** |
| Age | 1.1 | 0.98 - 1.29 | 0.151 |
| NYHA Class 4 | 0.92 | 0.06 - 24.94 | 0.951 |
| INTERMACS Score 2 | 2.96 | 0.42 - 26.46 | 0.282 |
| Baseline eGFR | 1 | 0.96 - 1.03 | 0.939 |
| Pre-op levosimendan | 0.45 | 0.05 - 4.04 | 0.456 |
| Multivariate general logistic regression model on determinants associated with right ventricular failure.  CI=confidence interva; HR=hazard ratio; eGFR= estimated glomerular filtration rate, using MDRD equation | | | |
